# Differences in the functional use of two migratory stopovers by humpback whales (*Megaptera novaeangliae*)

**DOI:** 10.1371/journal.pone.0321010

**Published:** 2025-05-14

**Authors:** Raphael Mayaud, Joshua N. Smith, David Peel, Craig Wilson, Wally Franklin, Tim Stevens, Susan Bengtson Nash

**Affiliations:** 1 Griffith School of Environment and Science, Griffith University, Brisbane, Queensland, Australia; 2 Harry Butler Institute, Murdoch University, Perth, Western Australia, Australia; 3 Data 61, CSIRO, Hobart, Tasmania, Australia; 4 Port of Brisbane Pty Ltd, The Port of Brisbane, Brisbane, Queensland, Australia; 5 The Oceania Project, Hervey Bay, Queensland, Australia; 6 Marine Ecology Research Centre, School of Environment, Science and Engineering, Southern Cross University, Lismore, New South Wales, Australia; 7 Australian Rivers Institute, Griffith School of Environment and Science, Griffith University, Brisbane, Queensland, Australia; University of Maryland Center for Environmental Science, UNITED STATES OF AMERICA

## Abstract

Humpback whale migration between tropical breeding grounds and polar feeding grounds is an energy-intensive activity undertaken on finite energy stores. The use of stopover sites to rest reduces energetic expenditure and provides enhanced opportunity for calves to nurse during migration. Moreton Bay is a newly identified migratory stopover for Australia’s east coast humpback whale population. Understanding the functional roles of stopovers is essential for a holistic understanding of population dynamics and connectivity. Therefore, contextualising the significance of Moreton Bay relative to a well-established stopover like Hervey Bay, can provide valuable insights into their functional roles within the broader migratory network, helping to inform targeted conservation efforts. To investigate this, we conducted a total of 865km of systematic, boat-based line transects across the two distinct geographical regions during temporally staggered periods (August and September – October) of the 2021 humpback whale migration. We examined population structure, behaviour, and habitat segregation, and developed spatial density surface models to predict density distribution patterns at each respective site. Our results show that Hervey Bay supports a more heterogenous mix of demographic groups, while Moreton Bay had a significantly greater number of calf-groups (z = 4.53, *p* = 0.017). Both bays exhibited similar resting behaviours, but social interactions among juveniles were unique to Hervey Bay. These findings suggest Moreton Bay serves a more utilitarian role as a stopover, functioning primarily as a resting site for mother-calf pairs, rather than the multifaceted use described in Hervey Bay. As lactating females and their calves are particularly vulnerable to anthropogenic threats like vessel strike, it is imperative to understand how different habitats contribute to the success of migration and ensure adequate protection is maintained.

## 1. Introduction

The broad spatio-temporal distributions of highly migratory species are a major challenge for spatial management [1]. Conservation plans tend to prioritise destination sites (*e.g.,* breeding and feeding) due to their functional significance, but often struggle to account for the spatial connectivity between them via migratory corridors [2]. This challenge can be further exacerbated when migratory pathways stretch over local, national, and international jurisdictions [3]. However, the repeated use of distinct areas along a migratory corridor can also play a fundamental role in the species life history and ecology. If such areas help in the recovery and persistence of a species, they are deemed to be critical habitats [4]. Today, there is growing recognition of the importance of understanding these critical habitats throughout a species’ migratory cycle to formulate cohesive management and policy measures [3]. Such information provides the legal basis for globally coordinated conservation measures such as those advocated by The Convention on the Conservation of Migratory Species of Wild Animals (CMS).

In addition, collaborative frameworks such as Ecologically or Biologically Significant Areas (EBSAs) identified under the Convention on Biological Diversity (CBD), Important Marine Mammal Areas (IMMAs) designated by the Marine Mammal Protected Areas Task Force, and Biologically Important Areas (BIAs) designated by governments globally are emerging as valuable approaches for delineating critical habitats and guiding conservation efforts. For highly migratory species, critical habitats include both destination sites and sites along migratory pathways, where animals temporarily aggregate to forage, socialise or rest [5]. These temporary aggregations are known as stopovers, and their significance have largely been documented among avian taxa (e.g., [6–9]). However, recent research has highlighted that transitory stopover sites are also common among baleen whales [10–16].

Humpback whales (*Megaptera novaeangliae*) perform one of the longest migrations on earth [17,18]. As capital breeders, they rely on accumulated energy stores acquired during intensive feeding in high latitudes to fuel their vast migration to tropical breeding grounds [17,19,20]. The migratory fast coincides with highly energetic reproductive investments such as male competitive breeding behaviours, and female gestation, parturition and lactation [21,22]. Accordingly, humpback whale migrations entail high metabolic expenditure which carry significant energetic costs to individuals [23,24]. For lactating females, accumulated energy stores must meet both their own energetic needs and those of a dependent calf. Humpback whales must therefore balance the energetic demands of migration via multiple physiological and behavioural adaptations, such as maintaining travel speeds that conserve energy [25], and optimising the amount of time they allocate to different activities such as resting [26]. Resting behaviour, which is often associated with nursing, is a particularly important energy conserving behaviour during migration [26,27] and can carry significant implications for calf fitness and reproductive success [23].

Convenient opportunities for rest and postpartum maternal care are characterised by warm, shallow, lagoon-like environments [14]. Warm waters may allow calves to invest greater energy into growth rather than thermoregulation [28], whilst shallow, sheltered waters, offer protection from predation and turbulent open ocean conditions, facilitating rest and nursing bouts [27]. Segregation in habitat-use by mother-calf groups typically into shallower waters also helps to avoid the aggressive attention of breeding adult males, which can increase energetic expenditure [29,30] and potentially lead to accidental mother-calf separation or calf death [31]. As a result, some habitats are preferred for resting and calving both within breeding sites and along migratory pathways. Examples of transitory stops along humpback whale migratory routes include the Kermadec [13] and Bermuda [12] Islands, the waters of São Tomé [32], oceanic features such as the ridges of the Rio Grande Rise [33] and seamounts in the South Pacific [34], and coastal embayment’s such as Exmouth, Jervis, Moreton, and Hervey Bay along Australia’s coastline [14–16,26,35–37].

Hervey Bay is the most documented stopover site for Australia’s east coast migrating humpback whale population, known as the E1 breeding stock [14,35,37,38]. This population has been on a rapid recovery trend since it’s near collapse during commercial whaling in the 20^th^ century [39]. The ecological and social significance of Hervey Bay to this population [40] is supported by high annual re-sighting rates [41] that are likely a reflection of maternally directed philopatry [35,42]. In contrast, Moreton Bay, an embayment ~300 kilometres south of Hervey Bay, has only recently emerged as an important stopover for the same migrating population [15]. Derived from systematic surveys spanning a five-year period, Castrillon et al. [15], documented an overriding seasonal presence of calf groups within the Bay during the southward migration that were frequently observed to be resting.

However, both embayment’s serve as prominent destinations for recreational boating and whale-watching activities [43–46]. Vessel traffic seeking close encounters with whales can lead to bioenergetic impacts such as decreased energy intake or increased energy expenditure [47] which is in turn known to impact individual stress physiology [48], reproductive success, and calf survival [49]. Furthermore, Moreton Bay experiences heavy commercial maritime traffic associated with the Port of Brisbane, the nation’s fastest-growing container port. Such activity poses significant threats to whales, including injury and death from ship strikes [50], as well as chronic disturbances like physical and behavioural disturbance and noise pollution [51–53]. Given the persistent and increasing levels of human activity within the region, and the potential significance of Moreton Bay to the E1 humpback whale population, further assessment of its role as a stopover is warranted.

As an extensively studied humpback whale stopover, Hervey Bay has previously served as a benchmark for understanding other humpback whale habitats along the east coast of Australia (e.g., [54,55]). Whilst previous studies have relied on comparing historical and opportunistic data, this study uses systematic, boat-based line transects performed in the two distinct geographical regions to contextualise the role of Moreton Bay within the broader migratory network. More specifically, the study aimed to investigate (1) population structure and behaviour, (2) density distribution, and (3) habitat segregation patterns, relative to the well-established stopover of Hervey Bay. Understanding habitat-use at multiple spatio-temporal scales is essential to anticipate conflicts with anthropogenic activity.

## 2. Methods

### 2.1. Study areas

**Fig 1:**
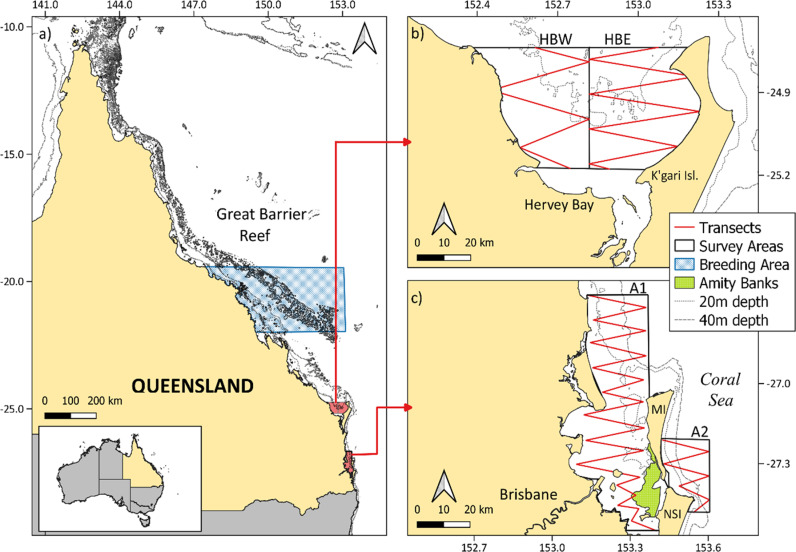
(a) Location of sampled survey areas (red shaded areas) relative to the Queensland coastline and core breeding area for the E1 humpback whale breeding stock (hashed blue area) and the line transect survey design for the (b) Hervey Bay survey region (i.e., two strata, HBW = Hervey Bay West (1527.12 km^2.^); HBE = Hervey Bay East (1749 km^2^)) and (c) Moreton Bay survey region (i.e., A1 = Moreton Bay (2284.9 km^2^); A2 = Coral Sea (438 km^2^)).

Hervey Bay is located ~600 km south of core breeding and calving areas for humpback whales identified in the Great Barrier Reef (GBR) (Fig 1) [19]. Bounded by the Queensland coast to the west and K’gari Island to the east, the largest opening to the Bay (~60 km wide) is to the north and connects the Bay to the Coral Sea [56] (Fig 1b). Aerial surveys conducted in 1988–1990 revealed humpback whale sightings were concentrated in the eastern part of Hervey Bay [57]. Studies on the abundance and distribution of humpback whales have since focused on this side of the Bay using non-systematic search approaches on platforms of opportunity such as whale-watching vessels [37] or dedicated research vessels [14]. To our knowledge, there has been no systematic survey covering the entirety of Hervey Bay since the aerial surveys flown in 1988. Yet, the E1 humpback whale breeding population has increased substantially [39], with consistent increases in the abundance of whales utilising Hervey Bay [41,58]. Density limitation of humpback whales in resting areas based on their behavioural space requirement is a potential effect of an increasing population [59]. As this may have altered the distribution dynamics of whales in Hervey Bay, this study aimed to survey an area representative of the aerial surveys performed in 1988. Due to the area’s large size, Hervey Bay was split into two strata to enable different search effort to be undertaken: Hervey Bay East (HBE) and Hervey Bay West (HBW). As available data indicate that whales are concentrated in the eastern portion of the Bay (e.g., [37,57]) transects were randomly placed in HBE using an equally spaced 5 km zigzag design to provide 100% coverage of the 1749 km2 survey area (Fig 1b). In HBW a 10 km equally spaced zigzag design was constructed to provide an 80% coverage of the 1527.12 km2 survey area. Transects were constructed using the survey design tool in Distance 7.3 [60].

Moreton Bay is located ~300 km south of Hervey Bay and its’ geological environment is comparable to that of Hervey Bay [61]. Moreton Bay is also bounded by the mainland to the west and large sand islands to the east (Mulgumpin and Minjerribah Islands; Fig 1c). These large sand islands are sister islands to K’gari possessing similarly impressive sand dunes and sand transport processes [61]. Two study areas were sampled in the Moreton Bay region: Area 1 (A1) encompassing Moreton Bay with a total area of 2284.9 km2 and Area 2 (A2) capturing a section of the main Coral Sea humpback whale migratory corridor east of Mulgumpin and Minjerribah Islands with a total area of 438 km2. Given transects in A1 and A2 were conducted during the same period, Area 2 provides a reference site to A1. This is because 90% of the humpback whale population passes within 5 km of the north-eastern headland of Minjerribah Island [39]. Sightings in Area 2 therefore reflects the migratory cohort in terms of relative abundance, group composition and behaviours. Equally spaced zigzag transects were designed to provide 100% coverage of each respective survey areas in the Moreton Bay region.

### 2.2. Data collection

#### 2.2.1. Timing of surveys.

Humpback whale migration behaviour is complex and involves inter- seasonal variations in the timing of the peak of the annual migration, as well as differences in the timing and migration rates among age, sex and reproductive classes [62–65]. Migration rates between Hervey Bay and Ballina, which is located 250km south of Moreton Bay, have been reported to range from 1.6 km/h to 3.5 km/h [65]. Consequently, it is expected that whales sighted in Hervey Bay may take on average anywhere from 3 to 8 days to reach Moreton Bay. However, different demographic groups are known to visit Hervey Bay for varying durations with the ‘whale-season’ lasting from July – October [35]. Peak whale abundance is nevertheless skewed towards the last two weeks of August [41]. By contrast, peak southward migration, when the predominant direction of travel is back towards feeding grounds in Antarctica, occurs along the main migratory corridor to the east of Moreton Bay from September-October [66]. To make a reasonable within-season comparison capturing the peak seasonal flow of whales passing through each respective Bay, transects were first performed in Hervey Bay during the end of August (23/08/2021–31/08/2021) followed by those in Moreton Bay eight days later. However, due to inclement weather in Moreton Bay and access to sections of the study area being less protected than in Hervey Bay, transects took longer to complete and commenced from mid-September (10/09/2021–07/10/2021).

#### 2.2.2. Sampling methodology.

Data collection protocol has been published elsewhere [15,50]. In brief, sightings of whales were recorded following line transect distance sampling methodology [67]. This method notes the angle and distance of the whale relative to the vessel’s centre line, vessel’s GPS location using the onboard Electronic Chart Navigation System, and the number of animals observed. Angles were measured using an angle board whilst distance were either estimated or measured when the vessel went off transect. Environmental conditions, such as sea state, wind speed and presence of fog, may affect the detectability of species and were recorded at the start of each transect, or whenever conditions changed. Transects were only conducted on days of optimal sea conditions (Beaufort Sea State of ≤ 4 and wind speeds less than 13 knots). When possible, fluke and dorsal photos were collected during surveys to rule out repeated sightings. Sightings made whilst the vessel was on transect were classified as “on-effort” and were used to model density distributions. Occasionally, the vessel broke off transect and went “off-effort” to collect more detailed observational data such as group composition and behaviour. Sightings made when the vessel was travelling to and from transects were also classified as “off-effort” data.

### 2.3. Humpback whale population structure and behaviour

Data with detailed observational information were used to describe the population structure and behaviour of humpback whales at both sites. These data were obtained when observers were able to confirm group composition, and when behavioural state was monitored for over 10 minutes. As such, this data was a mix of both on- and off-effort sightings. Slow vessel approaches were conducted to ensure there were minimal disturbances that could have led to changes in behaviour. Humpback whale group composition and behavioural terminology used are summarised in S1 and S2 Tables and follow similar classifications described elsewhere (e.g., [40,49,68]. Sightings that contained at least one calf were grouped as “calf groups” and sightings with no confirmed calves were grouped as “non-calf groups”. Behaviours were confirmed following these guidelines: when resting and logging lasted more than 15 seconds [49], when travelling was the predominant behaviour during the period of observation and when active behaviours like breaching and/or fin/tail slapping were repeated more than once. There was no lower time limit for apparent nursing behaviours, as successful nursing events from humpback whale calves can be short [49]. Behaviours associated to each sighting were used to classify them into five main behavioural categories (S2 Table) to capture important life-history functions. For example, behaviours that benefit conservation of energy budgets, such as resting, logging and apparent nursing were grouped into “energy-preserving” behaviours. Behaviours like breaching, tail-, and fin-slapping could be used to either classify the sighting into the “non-agonistic” behavioural group (if the group composition was made up of two or more whales, excluding mother and calves), or into the “other” category (if the sighting was just a singular whale or a mother-calf group; S2 Table). In addition to previous behaviours frequently documented, surveys of Hervey Bay revealed a unique social grouping, most accurately described as “sparring groups.” In such groupings, two or more whales showed some degree of inter-individual contact aggression, although lacking the high energy altercations associated with a competitive group. The behaviour resembled play-fighting and typically involved juvenile whales. For each of these sightings, the GPS location, date, time, depth (when it was noted using the onboard depth sounder) and predominant behavioural state was recorded.

### 2.4. Humpback whale density distribution

To predict humpback whale distributions in each survey area, a detection-adjusted density surface model (DSM) was built for each respective survey area [69]. This process involves a two-stage approach whereby a detection function is fitted to the on-effort transect data to calculate detection probabilities, and the second stage integrates this detection probability into a spatial model of abundance. The spatial model of abundance is constructed using a generalised additive model (GAM), with the response variable (the total count of whales per transect segment) modelled as the sum of geographical and informative environmental smooth functions to predict density over a unit area [69]. The area of each segment is included in the model as an offset term to account for relative survey effort.

#### 2.4.1. Detection function.

A detection function was fit to perpendicular distance data using the package ‘Distance’ in R [70] to estimate the probability of detecting an animal in relation to distance from the transect line [71]. Both Conventional (CDS) and Multiple Covariate Distance Sampling (MCDS) approaches were investigated. Half normal and hazard rate candidate models with and without cosine adjustment terms were first analysed before considering the addition of covariates that may influence the detection function. These covariates included Beaufort Sea State, wind speed, presence of fog, group size, and survey area. Survey area was categorised into Hervey (HBW and HBE) and Moreton (A1 and A2) due to sample sizes being insufficient to fit models to each individual area within each Bay. A right-truncation of the data was applied that was equivalent to the distance at which the probability of detection dropped to approximately 0.15, as recommended by Buckland et al. [67].

Detection functions were estimated for ungrouped data (e.g., stratified by geographic survey area) and pooled data (combined across geographic strata). Grouping data together can improve detection function if the ability to detect whales does not significantly differ between surveys. In this study, a 7m monohull research vessel, observation team and line transect methodology were kept constant across all survey areas, and it was assumed that the ability to detect whales did not differ greatly. This was further explored by fitting hazard-rate and half-normal detection function models that were (1) pooled together and (2) modelled with survey area included as a covariate factor and then examining the resulting Akaike Information Criterion (AIC) scores and Q-Q plots. All models that showed a significant model fit (*p* > 0.05) following a Cramer von-Mises goodness-of-fit were compared. The final model was then selected based on the model with the lowest AIC score.

#### 2.4.2. Density surface modelling (DSM).

***Environmental covariates*:** Environmental covariates considered in candidate DSMs included sea surface temperature (SST), depth and distance from shore, with the latter being split into distance from the mainland (dist.qld) and distance from islands (dist.isl). These covariates were selected based on being the top three most common environmental driver categories used to investigate humpback whale distributions [72] and their demonstrated biological relevance to humpback whales on their breeding and calving grounds [19,73]. Depth was obtained from the GEBCO bathymetry Grid-database on a 15 arc-second interval grid whilst SST was extracted from a daily NOAA dataset at a 1 km spatial resolution. Average SST was estimated per 1x1 km grid cell for each survey areas during the month of August (in Hervey Bay) and September and October (in Moreton Bay) to reflect SST patterns during the period of data collection. Distance from shore was calculated as the shortest distance between the midpoint of each transect and the nearest point of shoreline using the “*Distance to nearest hub*” tool in the QGIS Analyst toolbox [74].

Given the primary aim was to model humpback whale distribution and abundance across defined surveyed geographic areas and not predict beyond these sampled areas, we also included smooths of spatial location within the GAMs. Spatial location was transformed from latitude and longitude to metres north (y) and east (x) from the centre of the region of interest. Spatial smooths account for spatial effects that are not captured by other environmental covariates. However, as environmental covariates often vary in space, the bivariate smooth of location can be highly correlated with other environmental drivers. Multiconcurvity in covariates can mask a substantial part of the predictive power of other variables, thus making it difficult to separate the effects of the different variables. Multiconcurvity and collinearity between covariates were tested for each candidate GAM by using the functions *dsm.cor* and *vis.concurvity* in R packages “*dsm*” and “*mgcv*” respectively [75,76].

***Modelling*:** Surveyed line transects were divided into segments of ~ 5 km and environmental covariates were matched to the geographical midpoint of each segmented transect. Transects were split into 5 km segments as this was at least twice the truncation distance (2*w*) [69]. Sightings were summed for each segmented transect and a total effective strip width area (ESWA) was estimated by multiplying the segment area *A* (i.e., the product of its effective strip width, 2*w*, and its segmented length) by the probability of detection (P_a_) derived from the detection function model. The response variable (per segment total whale counts) was then modelled as a function of the environmental and spatial covariates using a GAM (assuming a log link function).

Models were fitted using thin-plate regression splines. The maximum basis size of smooth terms were limited to 10 for univariate terms and 20 for bivariate terms. Both univariate (s(x)+s(y) and bivariate (s(x,y) smooths of location were investigated in models. Models were initially built with all available covariates and then a shrinkage approach [77] was used to remove non-significant (*p > *0.05) terms. We used the double penalty shrinkage approach (e.g., by the select = TRUE function in “*mgcv*”) because it penalises the spline basis that affects functions in the range space as well as an additional penalty that affects functions in the penalty null space [77]. This process shrinks linear effects back to zero effects, allowing the removal of such terms from the model in a single step and without the problem of path dependence. If covariates demonstrated a linear behaviour (i.e., possessing an estimated degrees of freedom value of 1 in the model summary), models were re-run but with the covariate added as a linear term. Final GAMs were selected based on models with remaining significant covariate terms that demonstrated best model fit. This was based on models that showed low AIC values, high explained deviance, and demonstrated good model fit when assessing model diagnostics, such as Q-Q plots of deviance residuals and plots of random quantile residuals versus the linear predictor. We used the restricted maximum likelihood (REML) optimization method [76] and assumed a tweedie response distribution. The tweedie response has an advantage of handling zero inflated data in a unified way [78]. Since there was no covariance between the detection function and spatial model, the *dsm.var.gam* function was used in the R package “*dsm*” to calculate the uncertainty and confidence intervals of abundance estimates [75].

### 2.5. Humpback whale habitat segregation

A key observation on humpback whale breeding, calving and resting areas is the distinct habitat segregation according to demographic context. For instance, mother calf pairs are often present in shallow waters close to the coast, while other adult groups are more widely distributed [30,73,79]. To test whether there were significant differences in depth and distance to shore between calf and non-calf groups, basic hypotheses tests were performed [30]. Humpback whale sightings in Hervey Bay (HBW and HBE combined) and Moreton Bay (A1) where group composition was known were matched to depth by overlaying their GPS location with depth values in QGIS. The shortest distance to shore was then calculated for these whale sightings as has previously been described above. Data were tested for normality using the Shapiro-Wilk test and either Welch Two Sample T-tests or Wilcoxon rank sum tests were performed accordingly. To test whether a calf group was more likely to undertake energy-preserving behaviours either a Chi-squared test of independence or Fisher’s Exact test was used depending on the sample size (e.g., a Fisher Exact test was performed if sample size was low). Both these tests are suitable to assess associations within categorical data. To provide a direct comparison of the proportions of calf groups between the two sites, a test for equal proportions was conducted. This statistical test determines whether the proportions of a particular characteristic are significantly different between two independent groups. In this case, whether the probability of encountering a calf group was significantly different across Hervey and Moreton Bay.

## 3. Results

Transects were first completed over 8 days in Hervey Bay during the end of August (23/08–31/08) and then over 13 days in Moreton Bay from mid-September to early October (10/09–07/10). A total of 864.63 km of transects were performed during the field season, covering 399.86 km in Hervey Bay and 464.77 km within the Moreton Bay region (Table 1). A total of 126 sightings comprising 248 individual whales were made across the two survey areas: 76 sightings of 149 whales were observed in Hervey Bay compared to 50 sightings of 99 whales in Moreton Bay (Table 1).

**Table 1:** Summary of humpback whale sightings respective to each survey area and sampled strata. All data (on and off effort sightings) and truncated data (on effort sightings data following a 5% truncation distance for spatial modelling).

Survey Region	Strata	All data		Truncated data		
		Sightings	Individuals	Transects (km)	Sightings	Individuals
Hervey Bay	HBE	73	145	260.12	39	71
	HBW	3	4	139.73	3	4
Moreton Bay	A1	26	50	377.39	24	46
	A2	24	49	87.38	13	25

### 3.1. Humpback whale population structure and behaviour

**Fig 2:**
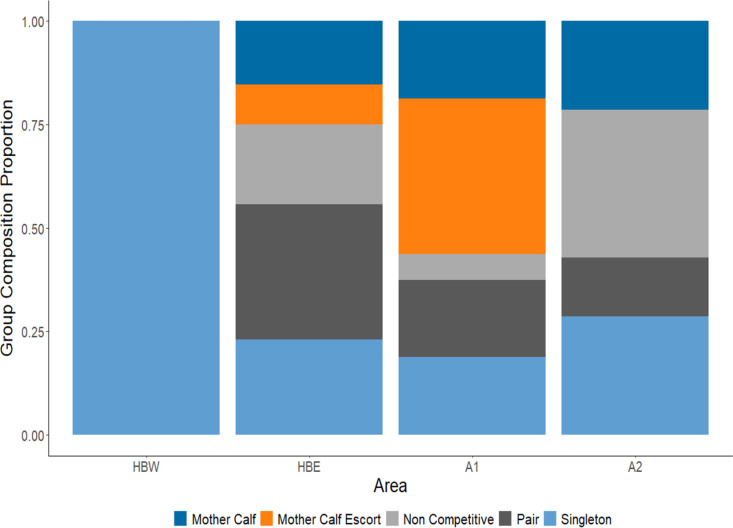
Stacked bar plot showing the proportion of group composition in each survey area. HBW = Hervey Bay West (1527.12 km^2.^); HBE = Hervey Bay East (1749 km^2^); A1 = Moreton Bay (2284.9 km^2^); A2 = Coral Sea (438 km^2^). Refer to S1 and S2 Tables for definitions of each composition. Surveys in Hervey Bay were conducted in August and surveys in Moreton Bay were conducted in September – October.

Of the 126 sightings, a total of 84 sightings had confirmed group compositions: 64% (n = 54) of those sightings were collected in Hervey Bay, and 36% (n = 30) in Moreton Bay. Group composition in HBE (n = 52) showed the greatest variation of all surveyed areas (Fig 2) but was mostly dominated by pairs (32.69%; n = 17) followed by singletons (23.08%; n = 12), non-competitive groups (19.23%; n = 10), mother-calf pairs (15.38%; n = 8) and mother-calf escorts (9.62%; n = 5). Only two sightings of known group composition were recorded in HBW, which were both single whales. In total, the number of calf groups in Hervey Bay made up 25% (n = 13) of the sightings and of those 62% (n = 8) were unescorted calf-groups.

The number of sightings with a calf present were significantly greater in A1 than in Hervey Bay following a two-sample test for equality of proportions (z = 4.53, *p* = 0.017). The proportion of calf groups in A1 totalled 56% (n = 9); 67% (n = 6) had an accompanying escort whilst 33% (n = 3) were unescorted mother-calf groups. An equal number of singletons, pairs, and mother-calf pairs were observed in A1 (19%, n = 3), whereas only one sighting of a non-competitive group (6%) was recorded (Fig 2). Area 2, which represented the main Coral Sea migratory corridor, had the highest percentage of non-competitive groups in all survey areas (36%, n = 5) (Fig 2). Single whales were the next common group type for this area (29%, n = 4), followed by mother-calf groups (21%, n = 3) and lastly pairs (14%, n = 2). No mother-calf escort groups were recorded in this area at the time of survey.

**Fig 3:**
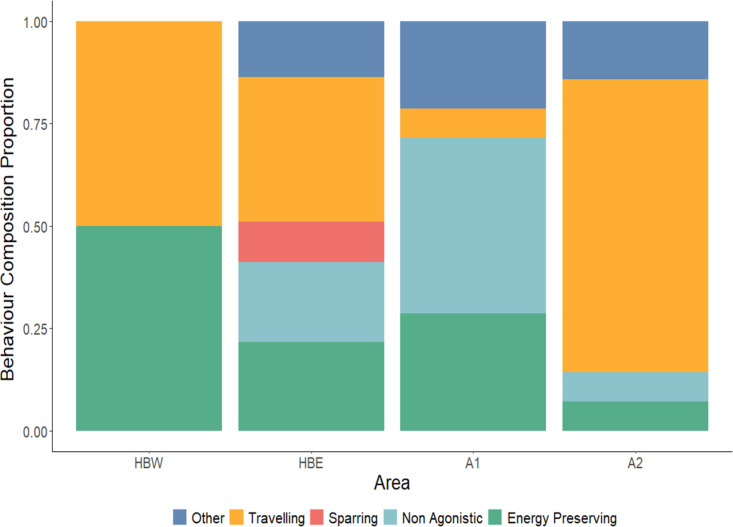
Stacked bar plot demonstrating the proportion of behaviour observed within each surveyed area. HBW = Hervey Bay West (1527.12 km^2.^); HBE = Hervey Bay East (1749 km^2^); A1 = Moreton Bay (2284.9 km^2^); A2 = Coral Sea (438 km^2^). Refer to S1 and S2 Tables for definitions of each behaviour grouping. Surveys in Hervey Bay were conducted in August and surveys in Moreton Bay were conducted in September – October.

Confirmed behavioural data were collected for 81 of the 84 known group composition sightings and provided the following calculated behavioural data percentages. Of all survey areas, *sparring groups* were only recorded in HBE and accounted for 9% (n = 5) of observations (Fig 3). *Energy-preserving* behaviours accounted for 22% (n = 11) of the sightings in HBE, where over half (54.5%) of those sightings had a calf present. *Non-agonistic* behaviours were recorded in 20% (n = 10) of sightings, whereas 35% (n = 18) of sightings were observed to be *travelling* whales and the remaining 14% (n = 7) of behaviours were recorded to be in the *other* category. Of the two whales with recorded behaviour in HBW, one was logging, and the other was traveling towards HBE (Fig 3).

The most common behaviour class in A1 was non-agonistic social behaviours (43%; n = 6), with the majority (67%) of group types in this behaviour class composed of mother-calf escorts breaching or fin slapping. A slightly higher percentage (29%; n = 4) of whales in A1 were observed to be undertaking energy-preserving behaviours compared to HBE. Area 2 was dominated by whales travelling (71%; n = 10), consistent with the fact this area represents the main Coral Sea migratory corridor. Across all survey areas, calf-groups were more likely to be undergoing energy-preserving behaviours than non-calf groups (*X*^2^ (1, N = 81) = 11.55, *p* = 0.0007). However, there was not a statistically significant association between energy-preserving behaviours and whether a calf group was escorted or unescorted (Fisher’s Exact Test, *p* = 0.6887).

### 3.2. Humpback whale density distribution

Following a 5% truncation distance, on effort sightings available for spatial modelling varied across survey areas (Table 1). A half normal detection function (weighted Cramer-von Mises test, *p* = 0.382) with no covariates or adjustment terms was selected. Although AIC favoured the model with fog as a covariate, there were substantially fewer sightings in the category where fog was recorded compared to the category where no fog was present. As both models conformed well to one another (within 2 AIC points) we opted to choose the most parsimonious model. The top three GAM models for each respective areas with their associated significant terms and AIC value are presented in Table 2.

**Table 2:** Summary of the top three generalised additive models (GAMs) to explain the distribution of humpback whales in each respective study site based on Akaike Information Criterion (AIC). The final, selected model used in predicting humpback whale distribution are highlighted in bold. x and y represent the easting and northing in kilometres from the centre of the study area. CI = 95% Confidence Intervals of estimated abundance and CV = Coefficient of variation for the final, selected spatial model.

Response Distribution	Terms	ΔAIC	Deviance Explained	Abundance	95% CI	CV
Hervey Bay						
**Tweedie (p = 1.141)**	**x, s (Depth)**	**0**	**31.02%**	**214**	**148–309**	**0.19**
Tweedie (p = 1.141)	s (x,y)	1	31.01%	219	151 - 316	
Tweedie (p = 1.142)	s (Dist.Isl), s (Dist.qld)	1	30.91%	211	148 - 310	
A1						
**Tweedie (p = 1.135)**	**s (x,y)**	**0**	**42.78%**	**102**	**61 - 169**	**0.263**
Tweedie (p = 1.133)	s (x,y), s (Depth)	1	45.63%	105	63 - 175	
Tweedie (p = 1.132)	s (x,y), s (SST)	2	43.65%	103	62 - 170	
A2						
**Tweedie (p = 1.01)**	**s (x,y)**	**0**	**71.58%**	**53**	**34–83**	**0.229**
Tweedie (p = 1.01)	s (x,y), s (Dist.Isl)	9	70.18%	59	35 - 99	

**Fig 4:**
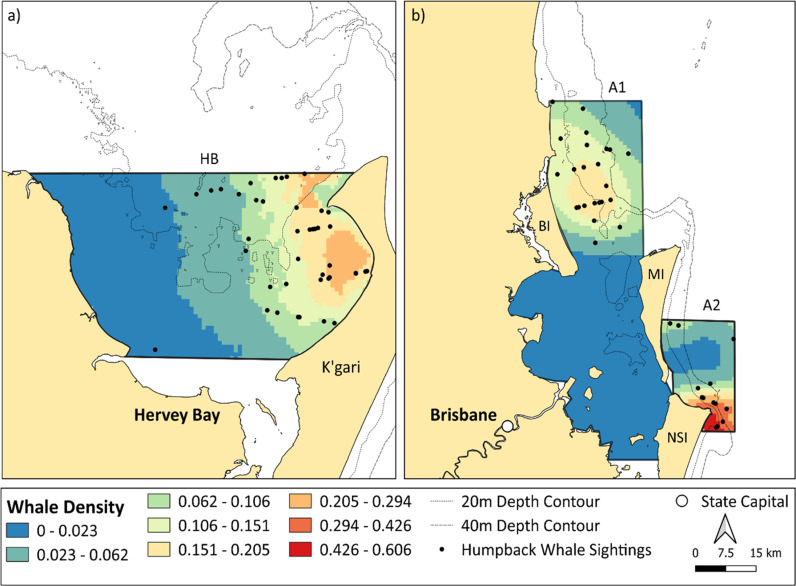
Humpback whale predicted densities for (a) Hervey Bay survey area, incorporating both strata (HBE and HBW) and (b) Moreton Bay survey area representing both A1 and A2. MI = Mulgumpin Isl. BI = Bribie Isl. and NSI = Minjerribah Isl. Scale is whale density as individuals per km^2^. Surveys in Hervey Bay were conducted in August; surveys in Moreton Bay were conducted in September – October.

The DSMs explained 31.02% (Hervey Bay), 42.78% (A1) and 71.58% (A2) of the deviance (Table 2) and predicted the highest densities of humpback whales close to Point Lookout in A2 (Fig 4). In Hervey Bay, a combination of a smooth of longitude and depth as covariates gave the best model fit. Whale density increased with longitude as evidenced by the smooth of plots of longitude (S1a Fig). As such, the DSM predicted the highest densities of humpback whales toward the eastern side of the Bay (Fig 4a). The highest densities of whales occurred between 15–25 m depth (S1b Fig). The estimated humpback whale abundance in the Hervey Bay survey area was over twice as much as in A1, with 214 whales (CV = 0.19, 95% CI = 148–309) compared to 102 whales (CV = 0.263, 95% CI = 61–169) (Table 2). Note, the study area size does differ between these two areas, and this should be considered when interpreting these results. In Moreton Bay, humpback whale density surface models were solely based on bivariate smooths of sighting locations. Despite being simpler, these models had the lowest AIC and demonstrated better model fit (Table 2). Humpback whale density was highest east of Bribie Island in A1, and distribution of whales seemed to follow the Queensland coast and across the mouth of Moreton Bay towards Mulgumpin Island. A high density of whales was captured off Point Lookout, the easternmost headland of Minjerribah Island in A2 (Fig 4b).

### 3.3. Humpback whale habitat segregation

**Fig 5:**
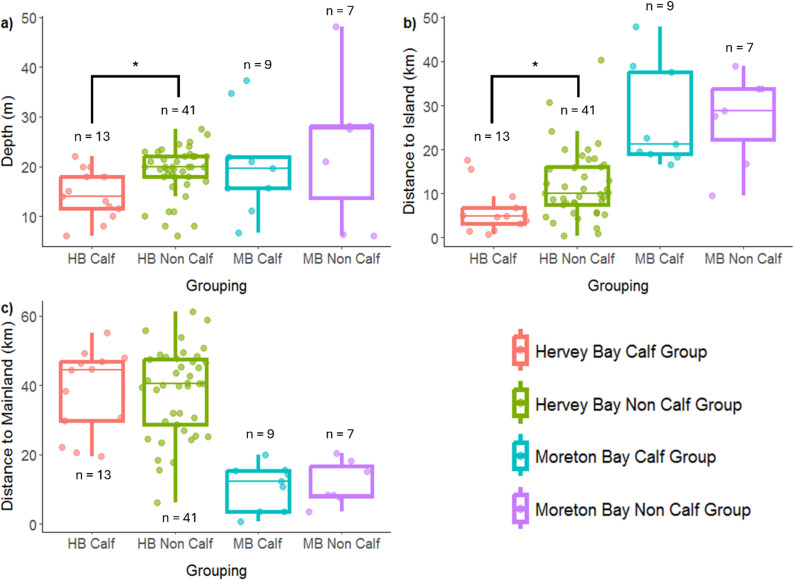
Habitat characteristics of calf and non-calf groups for Hervey Bay and Moreton Bay (A1). (a) Depth, (b) Distance to Island(s) and (c) Distance to mainland Queensland. Box and whisker plot shows the median value, and 25^th^ and 75^th^ percentile ranges. Dotted values are datapoints with some jitter in the x-axis for ease of visibility. *p < 0.05 and n represents the number of sightings in each group.

In Hervey Bay, calf groups (n = 13) were found in significantly shallower water depths (median = 13.93 m) than non-calf groups (n = 41) (median = 19.88 m; Wilcoxon rank sum test: W = 136, *p* = 0.0074) (Fig 5a). Calf groups were also observed to be significantly closer to K’gari Island (median = 4.98 km) than non-calf groups (median = 10.12 km) (Wilcoxon rank sum test: W = 124, *p* = 0.0032) (Fig 5b). In contrast, depth between calf (mean = 20.41 m; n = 9) and non-calf groups (mean = 23.65 m; n = 7) in Moreton Bay did not significantly differ from one another following a Welch Two Sample t-test (*t* (10.25) = -0.5, *p* = 0.6264). Likewise, distance to islands were not significantly different between the two groups in Moreton Bay. Calf and non-calf groups did not differ significantly in terms of distance to the mainland in either Hervey or Moreton Bay (Fig 5c).

## 4. Discussion

The functional roles of migratory stopovers are numerous, and varied among migration systems, species and individuals [9]. They include sites that help migrants accumulate energy, aid physiological recovery, enable periods to rest, and socialise [5,9]. As a capital breeding migrant that seasonally undertakes migrations between feeding and breeding grounds on finite energy stores [80], humpback whales rely on the availability of stopover sites to facilitate energy accumulation (calf lactating from mother), energy conservation, and social and cultural convergence [13,26,38]. Therefore, the identification and protection of stopovers on the migratory corridor is critical for humpback whales. Moreton Bay has recently been proposed as an important stopover for the E1 population based on observations of aggregating whales, particularly resting mothers and calves [15]. Here, we improve our understanding of the function of this stopover and contextualise the population structure, behaviour, density and distribution of humpback whales using Moreton Bay with the well-established stopover, Hervey Bay. Our results highlight the flexible distribution patterns and functional roles of these two stopover habitats. Hervey Bay catered for a broader spectrum of pod groupings and behaviours, whereas Moreton Bay was dominated by calf groups. This work suggests Moreton Bay may act more as a utilitarian role as a resting stopover site, particularly for mothers and calves. In contrast, Hervey Bay likely serves more important social benefits as well as resting to a range of demographic classes.

The within-season element of this study and its staggered surveying periods was strategically designed with the aim of targeting approximately the peak of humpback whale abundance passing through each site. This consideration is important, as the number of humpback whales migrating to and from breeding grounds each year is aperiodic and appears to show a high degree of inter-annual variability, as some female humpback whales likely skip migration in some years [81,82]. By targeting these peaks, the study aimed to account for the potential annual variability in the demographic structure of the humpback whale population migrating along the east coast of Australia. In the experimental design and execution, it is important to consider that completion of transects in Moreton Bay took longer than Hervey Bay due to increment weather. This, combined with incomplete knowledge of the precise succession stage of migration captured at each geographical location, introduces some uncertainty for direct comparison of observations. Notably, the observation that the proportion of calf groups in Hervey Bay during this 2021 study (25%) was much lower compared to that previously recorded (*e.g.,* 40%; [14]) may be related to the fact mother-calf groups in Hervey Bay dominate from the end of August and peak in density by the end of September [14,40].

Nevertheless, this data represents the best available dataset and drawing from data published in previous long-term studies [14,15], the proportion of calf sightings in Moreton Bay consistently appears to be greater than in Hervey Bay. For example, Franklin et al. [14], demonstrate that over 13 years of sightings data, calves were present in 40% of sightings in Hervey Bay. In contrast, calves were consistently present in over 50% of sightings over five years of data in Moreton Bay [15]. Moreover, the difference in proportion of calf groups captured in this study, and previously by Castrillon et al. [15], between A2 and A1, reaffirms the importance of A1 to calf-groups. Area 2 represents the humpback whale migratory corridor, where 90% of the population passes through this area [39]. Our results show 21% of sightings in A2 were calf-groups compared to 56% in A1. This is similar to other studies along this migratory corridor, such as along the eastern coast of Jervis Bay, where 22% of migrating groups within the migratory corridor had a calf present [16]. The dominance of calf groups in A1 suggest an important role primarily for this maturational class.

Calf sightings in Hervey Bay were mostly unescorted (62%, n = 8), consistent with previous work [14,57]. In contrast, the majority (67%, n = 6) of calf sightings in A1 had an escort present, a finding that resembles breeding grounds reported in other parts of the world, such as in Hawaii [83]. Escorts are thought to be males either seeking conception of post-partum ovulating females [31,84], and/or protecting females and their calf from male harassment and predators [85]. Escorted mother-calf groups are more likely to incur higher energetic costs as swimming speeds have been shown to increase with increasing numbers of male escorts [30]. By extension, unescorted mother-calf groups may allow the mother to invest greater care to the calf [14]. In this study, we found no significant difference in the proportion of energy-preserving behaviours between calf groups and escorted calf groups, though the sample data for these statistics were small. This finding mirrors that documented by Cartwright and Sullivan [29] who found no difference in the reduced time spent resting between unescorted mother-calf groups and those accompanied by one escort. The proportion of escorted calf group sightings in Moreton Bay seems to fluctuate from year to year [15]. Combined with data presented here, such fluctuations and geographical differences may indicate differing male reproductive strategies at different stages of migration, or escorts acting as a “bodyguard” [86] in relation to external factors present in Moreton Bay that may be absent in Hervey Bay, such as increased male harassment [29]. More behavioural evidence is needed to explore these hypotheses further.

The social importance of Hervey Bay as a stopover where whales of differing maturational class interact and develop skills has previously been reported [38,40]. Although the range of behaviours between Hervey and Moreton Bay were quite similar, a key observation in Hervey Bay was the formation of “*sparring groups*”. The demographic of this group was prevalent mostly among smaller, juvenile whales. This captured behaviour likely reflect immature males and females benefitting from socialising opportunities with mature females [40] and supports the understanding that Hervey Bay functions as an important meeting point for different demographic groups where complex social behaviours occur [38]. These social interactions are thought to be important for the physical and social development of immature males and females [87]. By contrast, sparring groups were not observed in Moreton Bay. Instead, surface-active behaviours among calf-groups (*e.g.*, breaching calf) were the dominant behaviour. These behaviours are also important for calf development as they influence the rate of development of myoglobin stores which are important for marine mammals in achieving extended dive times [87].

Similarities in the proportion of energy preserving behaviours across the two bays illustrates the role of both bays as critical areas for nursing and resting. Mcculloch et al. [54], also describe the proportion of resting behaviours between Hervey Bay and Gold Coast Bay to be comparable. Furthermore, Jones et al. [16], report calf-groups in Jervis Bay spent ~35% of their time resting. Although we did not specifically measure the duration of time individuals spent in each behavioural state, the proportion of sightings in Moreton Bay that were resting at the time of observation was 29%. As each of these bays are located along the E1 migratory pathway, south of the putative breeding grounds in the GBR, they likely each represent calves at various ages of development and therefore size. The time spent by calves resting or logging in a resting area of the West Australian Breeding stock D has been shown to not differ with calf length [49]. Animal-borne tags have further demonstrated that suckling event duration does not vary much with the calves’ relative age [88]. Energy-preserving behaviours therefore likely remains important throughout the humpback whale migration, highlighting the potential conservation importance of sheltered marine embayment’s as stopover sites along the entirety of Australia’s east and west coastlines. This finding should encourage greater efforts into understanding the way we manage maritime disturbance in other similar marine embayment’s, not only in Australia but also along whale migratory corridors globally.

The skewed humpback whale distribution in Hervey Bay towards K’gari Island after more than 30 years between systematic surveys, supports previous non-systematic surveys [14,37] and establishes the importance of this island as a covariate in humpback whale habitat-use. K’gari Island offers protection from the easterly/south-easterly winds that are common during the austral winter period for the southeast Queensland coast. Calmer conditions close to the island allow calves to increase energy stores more efficiently by facilitating low effort suckling behaviour [27]. Shallower water depths near the island further benefit mother-calf groups by reducing male manoeuvrability, thereby mitigating unwanted male attention [30,89]. Our statistical analyses confirm spatial segregation exists according to demographic group in Hervey Bay, with calf groups observed significantly closer to K’gari Island and in shallower depths compared to non-calf groups. In contrast, no significant differences in depth or distance from shore were found between demographic groups in A1. Moreton Bay is a narrow survey area compared to Hervey Bay, and so it could be that there is not enough space for a significant spatial segregation pattern to occur. It could also be possible that no significant relationships according to demographic group was due to low sample sizes within Moreton Bay.

Despite the availability of similar environmental habitat to Hervey Bay, humpback whale habitat-use in Moreton Bay did not conform to the patterns observed in Hervey Bay. For example, whilst Mulgumpin Island also provides protection from the prevailing wind conditions, there were no humpback whale sightings within the sheltered embayment west of Mulgumpin Island. Further, modelling results did not confirm the preference of humpback whales for waters close to the island. Whilst some studies have shown similar humpback whale habitat use between different sites on a breeding ground [90], it is not uncommon for habitat selection to vary among regions [91]. Notably, variation in habitat-use can be driven by high levels of anthropogenic pressures [92,93]. For instance, humpback whale calf groups on a Hawaiian breeding ground were found to be distributed in deeper waters away from shorelines, likely because of high vessel activity [92,93]. In this study, the Port of Brisbane shipping channel in A1 organises high levels of commercial ship traffic to pass close to Mulgumpin Island. A southward humpback whale habitat shift into Moreton Bay has been forecasted to increase commercial ship strike risk [50]. Similarly, the western side of Mulgumpin Island is an area of intensive all-year tourism and recreational maritime traffic [43]. Recreational vessels contribute to ship strike [94] as well as disturbance effects which negatively impact the energetic expenditure between resting mother-calf groups. Associated noise pollution caused by high vessel activity can also lead to calf separation [27,53]. Contrary to Hervey Bay, it could be that the sheltered waters west of Mulgumpin Island may represent a source of disturbance that mother-calf groups would tend to avoid.

However, while no humpback whales were sighted within Moreton Bay during this survey, it is not uncommon to observe them entering the Bay [15]. For instance, surveys performed in 2017 and 2018 captured humpback whale sightings within the embayment itself (sub-area b of Fig 4; Castrillon et al. [15]). The inter-annual variability in Bay usage across years warrants further investigation but may be associated with humpback whales adapting their use of stopovers according to energetic status. Foraging success in capital breeders determines body condition which can fluctuate from year-to-year [23]. The Antarctic feeding grounds of southern hemisphere humpback whales experienced extreme climatic anomalies in 2017, which affected humpback whale prey availability [95]. Consequently, whales migrating in 2017/2018 were in poorer body condition [95]. These lower energy reserves may influence the increased utilisation of sheltered environments when migrating whales are in suboptimal body condition. Additionally, Brooks et al. [38], report that some humpback whales may skip Hervey Bay in certain years. It is therefore essential to consider stopover use within the broader context of the annual life cycle [9]. For example, previous life history events, such as poorer feeding conditions, may influence a migrant’s selection of a suitable stopover, as well as the number of stopovers it needs during a migratory cycle.

Beyond existing anthropogenic pressures in Moreton Bay, the contrast in functional roles between the two bays may additionally be influenced by the annual migratory return and site fidelity of whales utilising Hervey Bay [35]. The close association between calves and their mothers are critical for the learned behaviour that subsequently determines the choice of migratory route [42], destination [96] and stopover areas [35]. In instances of severe exploitation, such as whaling, this learned behaviour can either be lost through elimination, or abandonment of certain areas by individuals avoiding these pressures [97]. By extension, removal of the pressure may lead to a gradual return, even from few surviving maternal lineages. Mulgumpin Island accommodated the largest whaling station (Tangalooma) in the southern-hemisphere from 1952–1962 [98]. High whaling activity in the surrounding area during the mid-20^th^ century could have affected regional distributions, resulting in humpback whales choosing to avoid the Moreton Bay area. Female philopatry and site fidelity of humpback whales utilising Hervey Bay would have experienced an uninterrupted history of cultural transmission compared to ancestors with site fidelity associated with Moreton Bay. We strongly suggest future work should investigate any evidence for the annual return of humpback whales in Moreton Bay. This information could help distinguish whether Moreton Bay is a facultative stopover (i.e., one where mother-calf pairs only use based on their current energy needs) rather than a planned stopover like Hervey Bay where a core subgroup of humpback whales repeatedly return year after year.

### 4.1. Conclusion

Hervey Bay is a crucial social habitat for humpback whales and has previously been described as a “*Cavanbah*” [99]; a word defined by the Bundjalung People to describe a safe annual social meeting place. With a more equal proportion of group types and evident interactions between juvenile whales, this study lends support to the role that Hervey Bay is a comprehensive stopover where whales of differing demographic groups not only rest but also undergo active and social behaviours [38]. By contrast, dominated by calf-groups, we suggest Moreton Bay fulfils more of a utilitarian role, primarily for lactating females involved in maternal care. The variability in the functional roles of these two stopovers sites highlights the importance of understanding how different stopovers can support diverse, yet essential activities. Habitat degradation from increased anthropogenic activities, such as shipping, threatens the key characteristics that make these sites valuable, likely compromising the functions they may offer. Based on the apparent importance of Moreton Bay to calf-groups and their vulnerability to anthropogenic pressures, it is imperative to ensure adequate protection and management of this subgroup utilising Moreton Bay.

## Supporting information

S1 Table Definitions of terms used to classify humpback whale groups within this study. (DOCX)

S2 Table Definitions of terms used to classify humpback whale behaviours and behavioural groups within this study. (DOCX)

S1 Fig Generalized additive model response curves (solid lines) with 95% confidence intervals (shaded area) for the final Hervey Bay model a) linear term of longitude (x), represented as kilometres east of the centre of the study area and b) response of depth. The dashes along the x-axis indicates distribution of sampled values.
(DOCX)
