# Supplementary material for: Differences in the functional use of two migratory stopovers by humpback whales (Megaptera novaeangliae)
Source: PLoS One. 2025 May 14;20(5):e0321010. doi: 10.1371/journal.pone.0321010 (PMC12077682; doi:10.1371/journal.pone.0321010)
Supplement: S1 Table — (DOCX) [file pone.0321010.s001.docx]

**Table S1**. **Definitions of terms used to classify humpback whale groups within this study.**

| **Terms** | **Definition** |
| --- | --- |
| *Singleton* | One single humpback whale. |
| *Pair* | Two humpback whales (adult and/or juveniles) within one to two body lengths and moving in similar direction. |
| *Competitive group* | Three or more whales engaged in agonistic, “competitive” surface behaviours, which often consists of multiple males competing for access to a single focal female (1). |
| *Non-competitive group* | Three or more whales, excluding mother-calf escorts, within one to two body lengths and moving in a similar direction. |
| *Calf* | A whale born during the current breeding season, in close proximity to a large, adult whale, and visually estimated to be <70% of the body length of the adult whale (2,3). |
| *Mother-calf* | A calf, as defined above, in close association to the adult, assumed to be its mother (4). |
| *Mother-calf escort* | A mother-calf pair accompanied by a non-calf, non-yearling adult individual (4). |
