# Supplementary material for: Differences in the functional use of two migratory stopovers by humpback whales (Megaptera novaeangliae)
Source: PLoS One. 2025 May 14;20(5):e0321010. doi: 10.1371/journal.pone.0321010 (PMC12077682; doi:10.1371/journal.pone.0321010)
Supplement: S2 Table — (DOCX) [file pone.0321010.s002.docx]

**Table S2**. **Definitions of terms used to classify humpback whale behaviours and behavioural groups within this study.**

| **Terms** | **Definition** |
| --- | --- |
| *Travelling* | Whale moving in one direction at a relatively constant speed (5). |
| *Breaching* | Whale jumps (full or half breach) headfirst mostly clear of the water (2)*.* |
| *Fin-slap* | Whale slaps the surface of the water with its pectoral fin, ventrally or dorsally (2). |
| *Tail-slap* | Whale slaps the surface of the water forcefully with the ventral or dorsal side of its fluke (2). |
| *Resting* | When the whale is moving very slowly in no defined direction (5) |
| *Logging* | Whale remains completely at rest on the surface with no other behaviour visible for > 15 seconds (2). |
| *Apparent nursing* | When the calf dives below the peduncle of the mother to position its head near the mammary area and surfaces either side of the mother (6). |
| *Agonistic behaviours* | Aggressive surface behaviours typically exhibited in a competitive group of three or more whales (4). |
| *Sparring groups* | Two or more whales playfully competing / sparring with one another with no obvious fight for a female. |
| *Non*-*agonistic behaviours* | Two or more whales, excluding mother-calf pairs, involved in calm, low-energy actions with a lack of aggression (typical behaviours include spy-hops, rolling ventral side up, pectoral fin slapping, occasional breaching and lobtailing; (4). |
| *Energy-preserving behaviours* | Any group types that were resting/logging or a calf that showed signs of possible nursing. |
| *Other behaviours* | Behaviours that are neither competitive or non-agonistic as defined above and include whales that are predominantly travelling or breaching, lobtailing or pectoral slapping (4). |
