# Supplementary material for: Differences in the functional use of two migratory stopovers by humpback whales (Megaptera novaeangliae)
Source: PLoS One. 2025 May 14;20(5):e0321010. doi: 10.1371/journal.pone.0321010 (PMC12077682; doi:10.1371/journal.pone.0321010)
Supplement: S1 Fig — The dashes along the x-axis indicates distribution of sampled values. (DOCX) [file pone.0321010.s003.docx]

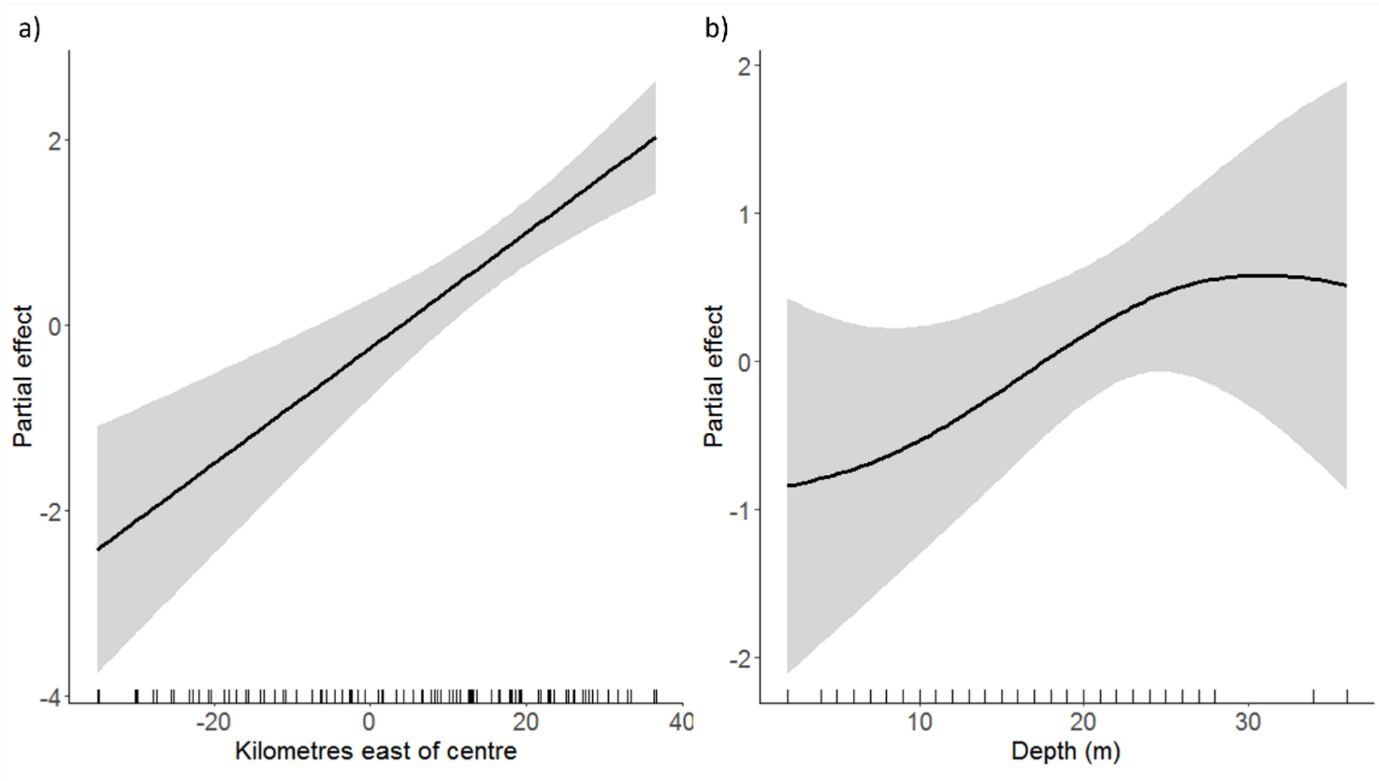


**Fig S1.** **Generalized additive model response curves** (solid lines) with 95% confidence intervals (shaded area) **for the final Hervey Bay model** **a) linear term of longitude** (x), represented as kilometres east of the centre of the study area and **b) response of depth.** The dashes along the x-axis indicates distribution of sampled values.
